# A global survey of neurosurgeons’ awareness of neural tube defect prevalence, prevention strategies, and their clinical time allocation to spina bifida care

**DOI:** 10.1007/s00381-025-06894-2

**Published:** 2025-07-18

**Authors:** Anastasia Arynchyna-Smith, Vijaya Kancherla, Inmaculada Aban, Alexander Arynchyn, Pedram Maleknia, David Becker, Andrzej Kulczycki, Jeffrey P. Blount

**Affiliations:** 1 University of Alabama at Birmingham, Birmingham, AL USA; 2 Emory University, Atlanta, GA USA

**Keywords:** Clinical time allocation, Neural tube defects, Prevalence, Prevention, Spina bifida, Workforce resources, Folic acid

## Abstract

**Purpose:**

Assess the global neurosurgeon workforce’s awareness of neural tube defect (NTD) prevalence, prevention strategies, and clinical time spent on spina bifida (SB) care.

**Methods:**

An online survey was administered to neurosurgeons worldwide between 2021-2022. Categorical responses were analyzed using descriptive analysis. Adjusted and unadjusted logistic regression analyses were conducted to examine the association between the clinical time spent on SB care and NTD prevalence awareness, controlling for sex, income region, pediatric training, and practice years, estimating odds ratios (ORs) and 95% confidence intervals (CIs).

**Results:**

Overall, 234 neurosurgeons (71% participation rate) responded from 64 countries. Respondents included 166 (77%) non-US residents practicing in non-high-income-country (non-HIC) (49%). Most were male (78%), 31-60 years old (80%), and urban-based (76%). Approximately quarter were unaware of the NTD prevalence at the national (28%) or global (24%) level, or the effectiveness of folic acid fortification (FAF) (27%). About 17% reported NTD rates ≥ 20/10,000 live births in their country. Neurosurgeons most often cited supplementation pills (46%) and flour FAF (36%) as the most common strategies to prevent NTD pregnancies. They were more likely to prefer strategies based on flour FAF (48%) and salt FAF (35%). There was a significant positive association between practicing in non-HIC and spending ≥ 20% of clinical time caring for SB (aOR = 6.42, 95% CI = 2.66-15.46, p < 0.001).

**Conclusions:**

This study highlights the need to improve awareness of NTD prevalence and preventive strategies among the neurosurgical community. There is a significant disparity in time spent on SB care between HIC and non-HIC countries.  Neurosurgeons can serve as key advocates for global and local SB prevention efforts, with particular emphasis on FAF.

**Supplementary Information:**

The online version contains supplementary material available at 10.1007/s00381-025-06894-2.

## Introduction

Globally, 214,000 to 322,000 live births are affected annually by neural tube defects (NTDs), including spina bifida (SB), anencephaly, and encephalocele [1]. Approximately 25% of pregnancies affected by NTDs are electively terminated, and another quarter result in stillbirths. Among live births, only 25% live past five years of age [1]. In low- and middle-income countries (LMICs), infections and hydrocephalus are the leading causes of mortality among newborns with NTDs [2–5]. Surviving infants with SB often face significant lifelong medical care (diagnosis, treatment, follow-up) and medical issues, including hydrocephalus, varying degrees of incontinence, and severe musculoskeletal and integumentary anomalies [4, 6, 7].

Until a few decades ago, patients with SB in LMICs rarely survived past adolescence [8]. However, survival rates in the United States and other high-income countries (HICs) have significantly improved: as of 2015, 92% of newborns survived past the first year, and 75% reached adulthood [4, 9, 10]. Despite these advancements, limited information is available on the proportion of neurosurgeons’ time dedicated to the ongoing care of patients with SB. In 2019, there were an estimated 35,622 (95% confidence interval (CI) [26,337, 44,908]) new neurosurgical operations related to newborn patients with SB worldwide [11]. This estimate, however, only reflects the surgical needs at birth, not the neurosurgical demands throughout the patient’s lifespan. Detailed time estimates for long-term neurosurgical care are critical for understanding the full neurosurgical needs of SB patients across their lifespan but still need to be developed. Such data could inform workforce training, resource allocation, and investments in the neurosurgical workforce.

Due to a shortage of neurosurgeons in LMICs, optimizing neurosurgical efficiency is essential for enhancing public health impact and maximizing the contribution of available expertise. Reducing the prevalence and severity of NTDs is a critical strategy for achieving this objective. Folic acid fortification is a proven, safe, and effective approach that significantly reduces NTD prevalence [12, 13]. However, only 68 of the 193 UN-recognized countries have any mandatory folic acid fortification policy as of 2022 [14]. Among countries that do fortify, there has been a significant reduction in the prevalence of NTDs to 10 per 10,000 live births on average [15–19].

The objectives of this survey-based study are three-fold. The first is to assess neurosurgeons’ awareness of SB prevalence in their respective countries and globally. Second, to examine neurosurgeons’ awareness of folic acid recommendations, primary prevention strategies in their countries, and the global status of fortification. Third, we conclude the study by estimating the amount of clinical time neurosurgeons dedicate to caring for patients with SB, with a particular focus on LMIC regions.

## Methods

### Survey development

The main body of the questionnaire consisted of 40 nominal or ordinal questions (33 multiple-choice, 6 True or False, one open text), several of which were used in an earlier survey of family physicians [20]. The primary author developed the remaining questions in collaboration with an epidemiologist specializing in birth defects and a senior pediatric neurosurgeon. The study team piloted with a local neurosurgery group the final version of the survey, which was then translated into six languages (English, Spanish, French, Hindi, Chinese, and Russian) by a nationally certified translation company. Survey questions focused on “neural tube defects” in the aggregate, with clinical time questions referring to “spina bifida” since other forms of NTD are less compatible with life. The Institutional Review Board at the primary author’s institution approved the study.

The first section of the survey measured neurosurgeons’ perceptions of their own country-specific NTD prevalence rate, global NTD prevalence rate, and overall NTD prevention rate following folic acid food fortification implementation. The second section assessed the knowledge gap regarding folic acid recommendations (primary and recurrence prevention). The third section assessed knowledge of current strategies for preventing NTD and additional potential folic acid fortification strategies from a neurosurgeon’s perspective. The fourth section assessed the neurosurgeon’s clinical time caring for patients with SB.

### Study design and sample

An online survey of practicing neurosurgeons worldwide was conducted, with respondents recruited using cluster and snowball sampling techniques. Cluster sampling focused on active members of the following organizations: International Society of Pediatric Neurosurgery, G4 Alliance, Foundation of International Education in Neurological Surgery, INCISION, African Society of Neurosurgery, American/Afghanistan women in neurosurgery, WhatsApp neurosurgical groups from Africa and Central/South America, American Association of Neurological Surgery. The snowball sampling technique was utilized. Eligible participants were neurosurgery residents, fellows, and faculty. Participants received a one-time email with a custom link to a questionnaire using REDCap software [21, 22]. Reminder emails were not sent due to requests from the neurosurgical organizations.

Per the World Federation of Neurological Surgery Global Neurosurgical Workforce database, there were 49,940 neurosurgeons globally in 2016. Most neurosurgeons worldwide (35,591, or 71%) are trained in just eight countries: China, Japan, the United States, India, Russia, South Korea, Brazil, and Germany [23]. Survey respondents were not compensated for their time. The survey was sent out over two months, open from October 2021 through October 2022, and took an average of 10 min to complete. The participation rate was calculated by dividing the number of respondents (those who submitted partial or complete answers) by the number of participants who opened the survey link. Valid percent is reported to identify variables that had missing values omitted. For comparison, respondents were stratified into three groups: low-income country (LIC), middle-income country (MIC), and high-income country (HIC) based on World Bank designation [24].

Definitions of terms used: underestimated global NTD rate (< 20 per 10,000 live births), current global NTD rate (20 per 10,000 live births), overestimated global NTD rate (> 20 per 10,000 live births); underestimated NTD rate with folic acid food fortification (FAFF) (0 per 10,000 live births), current NTD rate with FAFF (10 per 10,000 live births), overestimated NTD rate with FAFF (> 10 per 10,000 live births).

### Statistical methods

Descriptive data included participant representativeness per number of neurosurgeons per country and categorized into three income-group categories to protect respondents’ identity. The Clopper-Pearson method was used to calculate the exact confidence intervals of the proportions. Statistical significance was assessed using a p-value of 0.05. Fisher's exact test compared responses between non-HIC (LIC + MIC) and HIC regarding clinical care for patients with SB. Unadjusted and adjusted odd ratios (uORs and aORs) and 95% CIs were estimated using logistic regression analysis. The model assessed the binary outcome of < 20% and ≥ 20% of clinical time spent caring for patients with SB and the primary predictor of the country status (HIC or non-HIC), controlling for sex, HIC vs. non-HIC designation, formal training in pediatric fellowship, and years in professional practice based on the literature review selected a priori. The Pearson Chi-square test was used to compare responses between non-HIC and HIC regarding NTD prevalence awareness. The second model assessed the binary outcome of the knowledge gap of NTD prevalence in the respective country, present or absent, controlling for the same characteristics. This statistical analysis used the IBM SPSS Statistics (Version 29.0) package program.

## Results

**Fig. 1:**
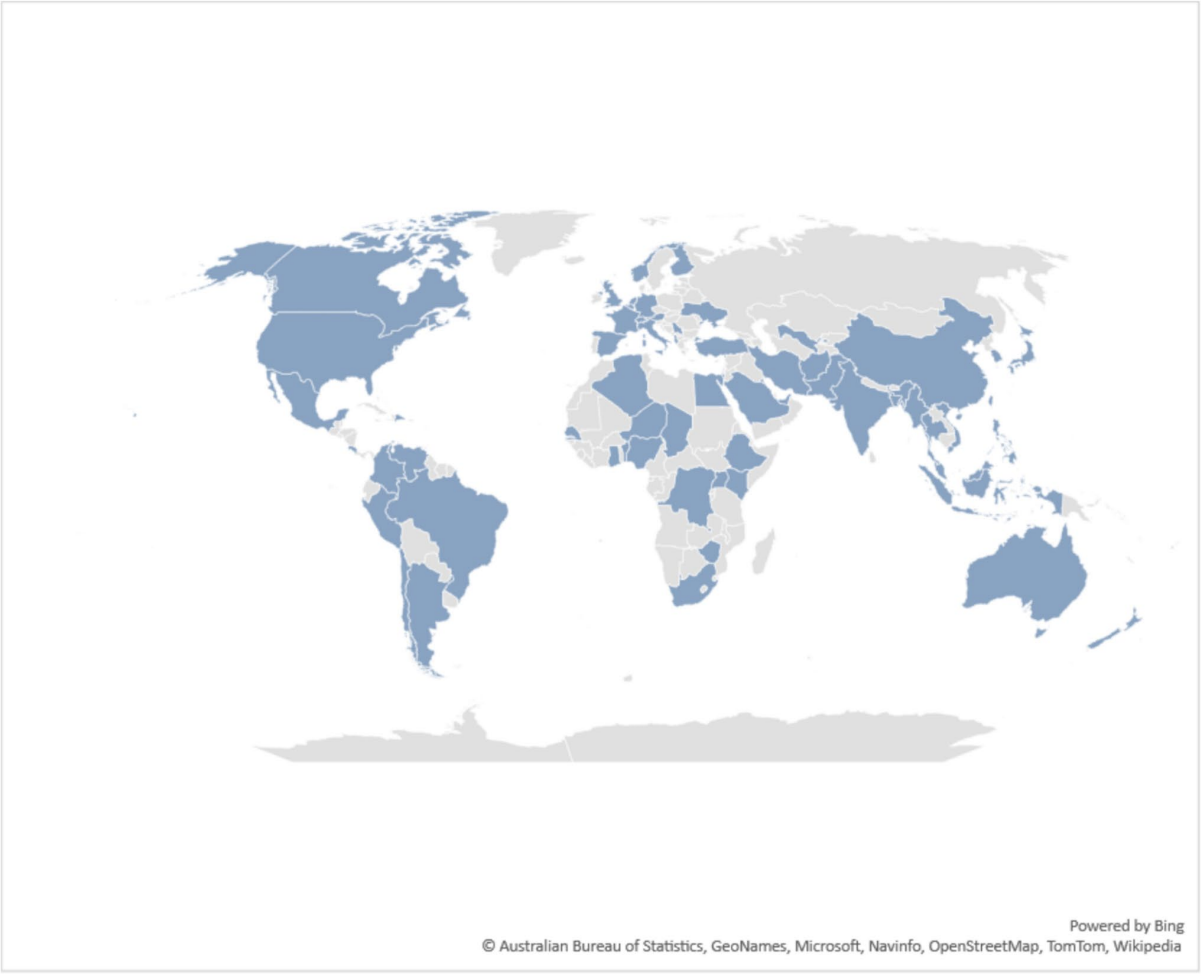
Representation of neurosurgery respondents (*n* = 234) by country (*n* = 65)

Overall, 234 (71%) of the 331 neurosurgeons who opened the survey link completed the study questionnaire. The demographic description of the respondents is summarized in Table 1. Respondents represented 65 countries and six continents, with 49% being from LIC and MIC. The majority were male (78.7%), had completed a formal pediatric fellowship (60%, from 18 HIC countries), and worked at a university-affiliated hospital in a large urban city (75.7%). One-third (33.6%) were in mid-career at 5–15 years in practice (Table 1, Fig. 1, Online Resource 1).

**Table 1:** Cohort characteristics (*n* = 234)

Variable	*n*	Percent
Age (in years) (*n* = 221)		
20–30	9	4.1%
31–40	63	28.5%
41–50	66	29.9%
51–60	48	21.7%
61–70	25	11.3%
71–80	9	4.1%
> 81	1	0.5%
Sex (*n* = 221)		
Male	174	78.7%
Female	47	21.3%
Completed formal pediatric neurosurgery fellowship (*n* = 220)		
Yes	132	60.0%
No	88	40.0%
Total number of years in practice (after residency) (*n* = 220)		
Still in training (residency)	19	8.6%
Still in training (fellowship)	6	2.7%
< 5 years	27	12.3%
5–15 years	74	33.6%
16–25 years	57	25.9%
> 25 years	37	16.8%
Current practice location (*n* = 218)		
Large urban city	165	75.7%
Small urban city	46	21.1%
Large rural city	3	1.4%
Small rural city	4	1.8%
Country of practice by income (*n* = 216)		
Low-income country (LIC)	15	7.0%
Middle-income country (MIC)	91	42.1%
High-income country (HIC)	110	50.9%

### NTD prevalence awareness

Overall, 28% of respondents reported being unaware (“Knowledge gap”) of the NTD prevalence in their country. Another 18% reported a high (≥ 20 per 10,000 live births) prevalence of NTDs (Table 2). Examination by income region indicated that LIC (45.5%) respondents reported a more extensive knowledge gap compared to those in MIC (29.1%) and HIC countries (25%). A higher percentage of HIC respondents reported lower NTD prevalence rates of 0 or 10 per 10,000 live births than those working in other regions (Online Resource 2). A statistically significant difference in reported NTD prevalence was found between HIC and other non-HIC regions (Online Resource 3). We investigated a subgroup of 56 respondents who reported a gap in knowledge of their respective NTD prevalence (Table 3). Both unadjusted and adjusted analyses showed formal training as a significant predictor of this knowledge gap. The adjusted model found that the odds of respondents without formal pediatric training having a knowledge gap were 3.66 (aOR 95% CI [1.79–7.79], p < 0.001) times that of the offs for those with pediatric training. Further, we found no significant interaction between country income designation (HIC vs non-HIC) and formal training in pediatric neurosurgery (p = 0.256) (Table 3).

**Table 2:** Awareness of the prevalence of neural tube defects (NTD) by the neurosurgical workforce

	Response (*n*)	Percent (95% CI)
**NTD rate per 10,000 live births (home country-specific to the respondent) (*n* = 205)**		
Not aware—"knowledge gap*"	57	27.8% (22%−35%)
None	20	9.8% (6%−15%)
10	91	44.4% (38%−52%)
20	23	11.2% (7%−16%)
50	10	4.9% (2%−9%)
100	4	2% (1%−5%)
**NTD rate per 10,000 live births (overall global) (*n* = 204)**		
Not aware—"knowledge gap"	48	23.5% (18%−30%)
None	1	0.5% (0%−3%)
10	56	27.5% (22%−34%)
20	61	29.9% (24%−67%)
50	24	11.8% (8%−17%)
100	14	6.9% (4%−11%)
**NTD rate per 10,000 live births (overall rate in countries that have a successful implementation of fortification of staple foods with folic acid) (*n* = 202)**		
Not aware—"knowledge gap"	55	27.4% (21%−34%)
None (underestimated)	31	15.4% (11%−21%)
~ 10 (the current rate at the time of the survey)	94	46.7% (40%−54%)
~ 20 (overestimated)	11	5.5% (3%−10%)
~ 50 (overestimated)	8	4% (2%−8%)
~ 100 (overestimated)	2	1% (0%−4%)

*NTD* neural tube defect

“Knowledge gap” is defined as not being aware of the national, global or post-fortification NTD rate.

**Table 3:** Logistic regression analysis of the respondent's knowledge gap of neural tube defects (NTD) prevalence in the respective country

		Knowledge gap present “Not aware”	Knowledge gap absent “Aware”	Unadjusted OR (95%CI)	*p*-value	Adjusted OR (95%CI)	*p*-value
Sex	Female	9 (15.8%)	32 (21.6%)	0.68 (0.3–1.53)	0.352	0.55 (0.22–1.37)	0.202
	Male	48 (84.2%)	116 (78.4%)	ref		ref	
Country's income designation	non-HIC	30 (53.6%)	67 (46.2%)	1.34 (0.72–2.5)	0.35	0.87 (0.43–1.76)	0.699
	HIC	26 (46.4%)	78 (53.8%)	ref		ref	
Formal training in pediatric neurosurgery	No	34 (59.6%)	45 (30.4%)	3.38 (1.79–6.38)	< 0.001*	3.66 (1.79–7.79)	** < 0.001^**
	Yes	23 (40.4%)	103 (69.6%)	ref			
Number of years in professional practice							0.396
	Still in training (residency)	5 (8.8%)	10 (6.8%)	3.88 (0.87–17.29)	0.076	1.78 (0.34–9.42)	
	Still in training (fellowship)	3 (5.3%)	3 (2%)	7.75 (1.15–52.3)	0.036*	3.94 (0.56–29.6)	
	< 5 yrs	8 (14%)	18 (12.2%)	3.44 (0.91–13.1)	0.069	3.28 (0.76–14.13)	
	5–15 yrs	21 (36.8%)	48 (32.4%)	3.39 (1.06–10.82)	0.039*	3.76 (1.09–13.0)	
	16–25 yrs	16 (28.1%)	38 (25.7%)	3.26 (0.99–10.77)	0.052	2.28 (0.79–9.79)	
	> 25 yrs	4 (7%)	31 (20.9%)	ref			

“Knowledge gap absent = Aware” is a reference category; *significant at *p* < 0.05

*Non-HIC* LIC and MIC, *LIC* Low-income country, *MIC* Middle-income country, *HIC* High-income country

Regarding their awareness of the global NTD rate, 23.5% of respondents reported no awareness, 28% underestimated it, and only 30% correctly estimated it as 20 per 10,000 live births (Table 2). Additionally, 27.4% of respondents were unaware of the NTD rate once effective food fortification with folic acid was implemented in a country. Only about half (46.7%:) correctly estimated it (Table 2).

### NTD prevention strategies

Current strategies most frequently reported by the respondents were supplementation pills (45.8%) and flour fortification with folic acid (36.1%) (Online Resource 4). However, responses varied across income groups, where neurosurgeons in HICs were more likely to report terminations. In contrast, “no prevention” was the most common response in LICs. The top four methods of NTD prevention endorsed by respondents were flour fortification (48.1%), salt fortification (35.2%), rice fortification (29.2%), and supplementation pills (29.2%). Salt was the top choice of potential vehicle for FAF for LIC respondents and the second choice in MIC and HIC countries.

Next, we assessed respondents’ awareness regarding global fortification efforts and barriers to global fortification. Half of the respondents answered that most LMIC regions do not fortify (50.9%), yet they expressed belief that fortification is a cost-effective strategy (46.3%). Similar responses were observed across all income regions. The most significant perceived barriers to global fortification were a lack of political will (52.8%) and a lack of awareness among politicians and consumers (52.3%).

### Clinical time allocation

In total, 43% of respondents reported spending 0% to 10% of their time caring for patients with SB, while 30% reported spending 11% to 20% (Table 4). A more granular analysis shows that, compared to wealthier countries, neurosurgeons in poorer regions spent more time caring for patients with SB noted by the darker shading gradient (Table 4). Both unadjusted and adjusted analyses showed that the odds of neurosurgeons working in non-HIC regions spending more than 20% of their clinical time on patients was 6.42 times more than the odds of their HIC peers (95% CI [2.66–15.46], p < 0.001) (Table 5).

**Table 4:**
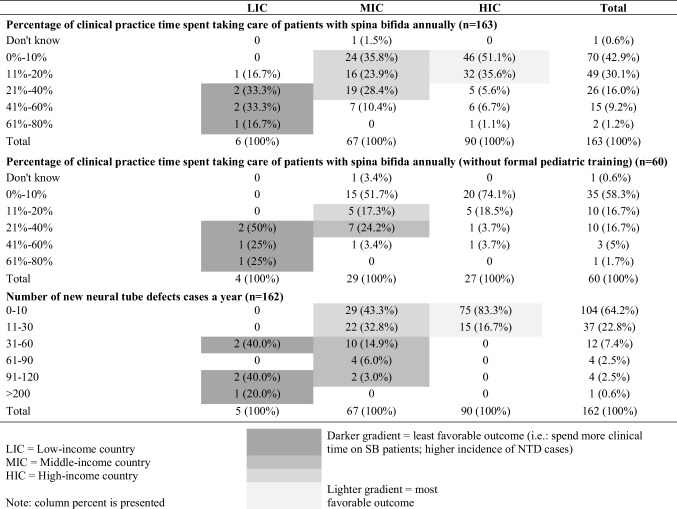
Clinical time spent on patients with spina bifida by the neurosurgical workforce

**Table 5:** Logistic regression analysis of the respondent's clinical time spent on patients with spina bifida annually

		< 20%	≥ 20%	Unadjusted OR (95%CI)	p-value	Adjusted OR (95%CI)^a^	*p*-value
Sex	Female	22 (18%)	10 (23.3%)	1.38 (0.59–3.21)	0.46	1.83 (0.70–4.78)	0.219
	Male	100 (82%)	33 (76.7%)	ref		ref	
Country's income designation	non-HIC	41 (34.5%)	31 (72.1%)	**4.92 (2.28–10.57)**	< 0.001*	**6.42 (2.66–15.46)**	< 0.001*
	HIC	78 (65.5%)	12 (27.9%)	ref		ref	
Formal training in pediatric neurosurgery	No	47 (38.5%)	14 (32.6%)	1.3 (0.62–2.71)	0.486	0.68 (0.29–1.58)	0.365
	Yes	75 (61.5%)	29 (67.4%)	ref		ref	
Number of years in professional practice	Still in training (residency)	10 (8.2%)	1 (2.3%)	0.34 (0.04–3.16)	0.345	0.15 (0.01–1.76)	0.131
	Still in training (fellowship)	5 (4.1%)	1 (2.3%)	0.69 (0.07–6.88)	0.748	0.48 (0.04–5.73)	0.559
	< 5 yrs	14 (11.5%)	8 (18.6%)	1.96 (0.58–6.57)	0.276	0.65 (0.16–2.61)	0.538
	5–15 yrs	39 (32%)	15 (34.9%)	1.32 (0.47–3.7)	0.599	0.55 (0.17–1.84)	0.331
	16–25 yrs	30 (24.6%)	11 (25.6%)	1.26 (0.42–3.74)	0.68	0.60 (0.17–2.09)	0.421
	> 25 yrs	24 (19.7%)	7 (16.3%)	ref			

< 20% clinical time spent is a reference category; *significant at *p* < 0.05.

^a^Adjusted for sex, income designation, formal training in pediatric neurosurgery, number of professional years in practice.

*Non-HIC* LIC and MIC, *LIC* Low-income country, *MIC* Middle-income country, *HIC* High-income country

Furthermore, 33% of respondents who practice adult and pediatric neurosurgery and did not have formal pediatric fellowship training estimated that they spent 11% to 40% of their clinical time on patients with SB. Only 15 (17%) of HIC respondents reported caring for more than 10 new NTD cases annually, compared to 43 (60%) of LIC and MIC respondents combined (Table 4). A darker shading gradient depicts the higher burden on the neurosurgical care in LIC and MIC regions.

## Discussion

To the best of our knowledge, this is the first paper to survey perceptions of NTD prevalence, prevention, and clinical burden from the viewpoint of neurosurgeons across the globe. Perceived prevalence varied significantly across countries. Neurosurgeons agreed that mandatory fortification of foods with folic acid is the most effective prevention method, as noted by earlier research [13, 14, 17, 19]. Consistent with higher rates of babies born with SB, neurosurgeons working in LMICs reported spending more clinical time caring for patients with SB.

The findings indicate the need for more awareness regarding the care burden of SB within the neurosurgical workforce and the extra challenges posed in resource-limited settings. Further, LIC respondents exhibited less understanding of the prevalence in the respective countries, although this issue should be addressed in all countries. The reported prevalence of NTD cases is greater in LIC and MIC, which tend to have less adequate neurosurgical resources to care for more patients. Some LIC and MIC respondents estimated they see between 61 and 200 cases a year, equivalent to a range of one new baby with NTD born every six days to every other day. Furthermore, adult neurosurgeons in non-HIC countries who had not completed a fellowship in pediatric neurosurgery appeared to spend more of their time on SB care than those in HIC countries. Overall, non-HIC neurosurgeons were significantly more likely to report spending over 20% of their time on SB care than their HIC colleagues. Dewan et al. (2019) provided very high estimates of the need for neurosurgical care at birth and stopped there due to a lack of follow-up data [11]. Our survey results complement the reported high neurosurgical needs at birth with post-birth estimates [11]. Adequate folic acid food fortification programs can prevent many of these cases [13]. However, with only half of the respondents being aware of the fortification's effectiveness, there is a clear need for education and improvement to increase prevention advocacy by neurosurgeons who see the daily struggles of this patient population. Our study provided insight from a direct patient care aspect that has not been studied in this medical specialty before.

Concerning prevention strategies, the survey showed that folic acid supplementation pills were still the most common prevention strategy in most countries, with folic acid flour fortification following suit. It was more prevalent in the wealthier regions. However, the data also show that HIC respondents were more likely to report offering termination of pregnancy as a strategy against NTDs. Respondents reported that lower-income regions had fewer prevention measures in place, contributing to greater healthcare needs. Neurosurgeons indicated a strong preference for food fortification – particularly of flour, salt, and rice – as the leading prevention strategy. Respondents across all income regions identified iodized salt with folic acid as the most practical first or second top choice for the prevention method of NTD. This shows that there may be good support for salt fortification with folic acid among the neurosurgical community. To the best of our knowledge, this is the first global survey to provide data on neurosurgeons’ perspectives on preventing NTD, and our data aligns well with the published literature on the effectiveness of and need to implement folic acid food fortification for such purpose [12, 13]. It is important to recognize that fortification as a preventive strategy can significantly reduce the prevalence of SB and alleviate the clinical burden on the neurosurgical workforce. This approach represents an optimal public health strategy at the population level.

## Limitations and strengths

Survey results are limited to neurosurgeons who responded, and their knowledge of the topics can only represent this subset of physicians. However, the survey achieved reasonably good global representation, with almost half of the respondents residing in non-HIC regions, representing 62% of countries classified as LMICs by the World Bank in 2023 [24]. The survey data provided helpful information for a very specialized field of medicine; the one most focused on caring for SB patients. As such, the findings provide a first-hand view of the neurosurgical community's awareness, knowledge, perceptions, and clinical time burden of SB.

## Conclusion

This survey highlights the need to improve awareness of the clinical burden that SB cases exert on scarce neurosurgical resources globally. Non-HIC neurosurgeons spend disproportionally more clinical time on patients with SB and have limited resources to provide adequate care. Such resources could be directed towards non-preventable conditions if most SB cases were prevented. There is a need to expand and improve educational outreach and surveillance to highlight the prevalence of NTD in each country. Neurosurgeons have a unique longitudinal vantage point to observe the suffering associated with SB across the lifespan, which makes them potential advocates for prevention through fortification with folic acid to reduce global disease burden.

## Supplementary Information

Below is the link to the electronic supplementary material.

Supplementary file1 (DOCX 29 KB)

## Data Availability

The data that support the findings of this study are not openly available due to sensitivity reasons and are available from the corresponding author upon reasonable request. Data are located in controlled access data storage at the University of Alabama at Birmingham.
