# Supplementary material for: A global survey of neurosurgeons’ awareness of neural tube defect prevalence, prevention strategies, and their clinical time allocation to spina bifida care
Source: Childs Nerv Syst. 2025 Jul 18;41(1):237. doi: 10.1007/s00381-025-06894-2 (PMC12274217; doi:10.1007/s00381-025-06894-2)
Supplement: Supplementary file 1 — Supplementary file1 (DOCX 29 KB) [file 381_2025_6894_MOESM1_ESM.docx]

**A global survey of neurosurgeons’ awareness of neural tube defect prevalence, prevention strategies, and their clinical time allocation to spina bifida care.**

*Child’s Nervous System*

Anastasia Arynchyna-Smith^1^, Vijaya Kancherla, Inmaculada Aban, Alexander Arynchyn, Pedram Maleknia, David Becker, Andrzej Kulczycki, Jeffrey P. Blount

^1^Division of Pediatric Neurosurgery, Department of Neurosurgery, University of Alabama at Birmingham, Birmingham, AL, USA, [arynch@uab.edu](mailto:arynch@uab.edu)

**Online resource 1** Response representation by World Bank income group (n=64 countries)

| Response representation rate | | INCOME | | | Total | |  |
| --- | --- | --- | --- | --- | --- | --- | --- |
|  |  | LIC | MIC | HIC |  | |  |
| <5% | Count | 1 (1.6%) | 22 (34.4%) | 22 (34.4%) | 45 (70.3%) | |  |
| <10% | Count | 1 (1.6%) | 5 (7.8%) | 2 (3.1%) | 8 (12.5%) | |  |
| <20% | Count | 1 (1.6%) | 4 (6.3%) | 1 (1.6%) | 6 (9.4%) | |  |
| >=20% | Count | 3 (4.7%) | 2 (3.1%) | 0 (0.0%) | 5 (7.8%) | |  |
| Total | Count | 6 (9.4%) | 33 (51.5%) | 25 (39.1%) | 64 (100%) | |  |
| LIC = Low-income country | | |  |  |  |  | |
| MIC = Middle-income country | | | |  |  |  | |
| HIC = High-income country | | |  |  |  |  | |

**Online resource 2** Neurosurgeon's awareness of country-specific prevalence of neural tube defects (NTD) by income group, n=201

|  | LIC | MIC | HIC | Total |
| --- | --- | --- | --- | --- |
| Not aware "knowledge gap" | 5 (45.5%) | 25 (29.1%) | 26 (25.0%) | 56 (27.9%) |
| 0 | 0 | 1 (1.1%) | 19 (18.3%) | 20 (10.0%) |
| 10 | 3 (27.3%) | 30 (34.9%) | 58 (55.8%) | 91 (45.3%) |
| 20 | 2 (18.2%) | 20 (23.3%) | 1 (1.0%) | 23 (11.4%) |
| 50 | 0 | 9 (10.5%) | 0 | 9 (4.5%) |
| 100 | 1 (9.1%) | 1 (1.1%) | 0 | 2 (1.0%) |
| Total | 11 (100%) | 86 (100%) | 104 (100%) | 201 (100%) |
| Footnote: NTD rates not validated against reported estimates from surveillance systems | | | | |
| NTD = neural tube defect |  |  |  |  |

|  |  |  |  |  |
| --- | --- | --- | --- | --- |
| LIC = Low-income country |  |  | Darker gradient = least  favorable outcome | |
| MIC = Middle-income country | |  |  |  |
| HIC = High-income country | |  |  |  |
| Note: column percent is presented |  |  | Lighter gradient = most  favorable outcome | |

**Online resource 3** Neurosurgeon's awareness of the country-specific prevalence of neural tube defects (NTD) by income groups: 3 categories

|  | Non-HIC | HIC | Total |
| --- | --- | --- | --- |
| Knowledge gap of NTD prevalence is present | 30 (30.9%) | 26 (25%) | 56 |
| 0-10 NTD per 10,000 live births | 34 (35.1%) | 77 (74%) | 111 |
| 20-100 NTD per 10,000 live births | 33 (34%) | 1 (1%) | 34 |
| Total | 97 | 104 | 201 |
| Pearson chi-square test, p<0.001 |  |  |  |
| NTD = neural tube defect  Non-HIC = LIC+MIC; LIC = Low-income country; HIC = High-income country | | | |
|  |  |  |  |

**Online Resource 4** Neural tube defects (NTD) prevention strategies from a neurosurgeon's perspective

|  | LIC (n=15) | MIC (n=91) | HIC (n=110) | Total (n=216) | | |  |
| --- | --- | --- | --- | --- | --- | --- | --- |
| Primary strategy in respondents' respective country (mark all that apply) | | | | | |  |  |
| Don't know | 0 | 1 (1.1%) | 5 (4.5%) | 6 (2.8%) | | |  |
| No prevention | 6 (40%) | 11 (12.1%) | 2 (1.8%) | 19 (8.8%) | | |  |
| No fortification of food with folate but universal health care, including screening and termination of affected fetuses, available to all pregnant women | 1 (6.7%) | 3 (3.3%) | 22 (20%) | 26 (12%) | | |  |
| Flour fortification with folic acid | 0 | 30 (33.0%) | 48 (27.7%) | 78 (36.1%) | | |  |
| Other food fortification with folic acid | 0 | 14 (15.4%) | 26 (43.6%) | 40 (18.5%) | | |  |
| Supplementation pills with folic acid are available at a low cost or no cost | 2 (13.3%) | 45 (49.5%) | 52 (47.3%) | 99 (45.8%) | | |  |
| Micronutrient supplement packets are available at a low cost or no cost | 0 | 5 (5.5%) | 15 (13.6%) | 20 (9.3%) | | |  |
| Depo-Provera birth control shot with folic acid available at low or no cost | 0 | 0 | 3 (2.7%) | 3 (1.4%) | | |  |
| No response | 6 (40%) | 0 | 0 | 6 (2.8%) | | |  |
| The most practical method to attain widespread food fortification with folic acid in your country (mark all that apply) | | | | | |  |  |
| Don't know | 0 | 1 (1.1%) | 7 (6.4%) | 8 (3.7%) | | |  |
| Promote daily multi-vitamin with iron and folic acid) | 1 (6.7%) | 19 (20.9%) | 30 (27.3%) | 50 (23.1%) | | |  |
| Mandatory fortification of flour with folic acid | 1 (6.7%) | 46 (75.4%) | 57 (51.8%) | 104 (48.1%) | | |  |
| Mandatory fortification of rice with folic acid | 1 (6.7%) | 28 (30.8%) | 34 (30.9%) | 63 (29.2%) | | |  |
| Mandatory fortification of iodized salt with folic acid | 6 (40%) | 31 (34.1%) | 39 (35.5%) | 76 (35.2%) | | |  |
| No cost folic acid supplemental pills to all women of reproductive age | 2 (13.3%) | 28 (30.8%) | 33 (30%) | 63 (29.2%) | | |  |
| Depo-Provera birth control shot with folic acid available at low or no cost | 1 (6.7%) | 5 (5.5%) | 6 (5.5%) | 12 (5.6%) | | |  |
| No response | 3 (20%) | 0 | 0 | 3 (1.4%) | | |  |
| Statements that best reflect the current situation regarding food fortification for prevention of NTD globally (mark all that apply) | | | | | |  |  |
| Folic acid fortification directly correlates with high Gross Domestic Product | 1 (6.7%) | 17 (18.7%) | 25 (22.7%) | 43 (19.9%) | | |  |
| Folic acid fortification is common worldwide | 0 | 14 (15.4%) | 6 (5.5%) | 20 (9.3%) | | |  |
| Most LMICs do not fortify food with folic acid | 3 (20%) | 43 (47.3%) | 64 (58.2%) | 110 (50.9%) | | |  |
| Folic acid fortification has significant health risks to the population | 0 | 2 (2.2%) | 1 (1%) | 3 (1.4%) | | |  |
| Folic acid fortification is not an effective method for prevention of NTD | 0 | 0 | 0 | 0 | | |  |
| Folic acid fortification is a cost-effective public health strategy for prevention of NTD | 4 (26.7%) | 35 (38.5%) | 61 (55.5%) | 100 (46.3%) | | |  |
| No response | 7 (46.7%) | 0 | 0 | 7 (3.2%) | | |  |
| The most significant barriers to attaining food fortification with folic acid of staple foods worldwide (mark all that apply) | | | | | |  |  |
| Don't know | 0 | 6 (6.6%) | 9 (8.2%) | 15 (6.9%) | | |  |
| Limited capacity among food producers to begin fortification | 1 (6.7%) | 21 (23.1%) | 14 (12.7%) | 36 (16.7%) | | |  |
| Limited flour production on a large scale | 1 (6.7%) | 10 (11.0%) | 7 (6.4%) | 18 (8.3%) | | |  |
| Too expensive for food producers | 1 (6.7%) | 12 (13.2%) | 16 (14.5%) | 29 (13.4%) | | |  |
| Lack of political will | 3 (20%) | 48 (52.7%) | 63 (57.3%) | 114 (52.8%) | | |  |
| Limited return on investment | 0 | 19 (20.9%) | 24 (21.8%) | 43 (19.9%) | | |  |
| Lack of awareness among policymakers and/or consumers | 5 (33.3%) | 46 (50.5%) | 62 (56.4%) | 113 (52.3%) | | |  |
| No response | 4 (26.7%) | 0 | 0 | 4 (1.9%) | | |  |
| Footnote: Column percentages may not add up to 100% since participants could have chosen more than one answer option | | | | | |  |  |
| LIC = Low-income country; MIC = Middle-income; HIC = High-income country country; NTD = neural tube defect; Note: column percent is presented | | | | | | | |
|  | |  |  |  |  | | |
|  |  |  |  |  |  | | |
|  |  |  |  |  |  | | |
|  |  |  |  |  |  | | |
